# Novel conjugates of aminoadamantanes with carbazole derivatives as potential multitarget agents for AD treatment

**DOI:** 10.1038/srep45627

**Published:** 2017-03-30

**Authors:** Sergey O. Bachurin, Elena F. Shevtsova, Galina F. Makhaeva, Vladimir V. Grigoriev, Natalia P. Boltneva, Nadezhda V. Kovaleva, Sofya V. Lushchekina, Pavel N. Shevtsov, Margarita E. Neganova, Olga M. Redkozubova, Elena V. Bovina, Alexey V. Gabrelyan, Vladimir P. Fisenko, Vladimir B. Sokolov, Alexey Yu Aksinenko, Valentina Echeverria, George E. Barreto, Gjumrakch Aliev

**Affiliations:** 1 Institute of Physiologically Active Compounds Russian Academy of Sciences, Chernogolovka, 142432, Russia; 2 Emanuel Institute of Biochemical Physics, Russian Academy of Sciences, Moscow, 119334, Russia; 3 Department of Pharmacology, Therapeutic Faculty, I. M. Setchenov Moscow Medical Academy, ul. Bol’shaya Pirogovskaya 2/6, Moscow, 119881 Russia; 4 Universidad San Sebastián, General Cruz 1577, Concepción, Chile; 5 Departamento de Nutrición y Bioquímica, Facultad de Ciencias, Pontificia Universidad Javeriana, Bogotá D.C., Colombia; 6 Instituto de Ciencias Biomédicas, Universidad Autónoma de Chile, Santiago, Chile; 7 GALLY International Biomedical Research Consulting LLC., San Antonio, TX 78229, USA; 8 School of Health Science and Healthcare Administration, University of Atlanta, Johns Creek, GA 30097, USA

## Abstract

A new group of compounds, promising for the design of original multitarget therapeutic agents for treating neurodegenerative diseases, based on conjugates of aminoadamantane and carbazole derivatives was synthesized and investigated. Compounds of these series were found to interact with a group of targets that play an important role in the development of this type of diseases. First of all, these compounds selectively inhibit butyrylcholinesterase, block NMDA receptors containing NR2B subunits while maintaining the properties of MK-801 binding site blockers, exert microtubules stabilizing properties, and possess the ability to protect nerve cells from death at the calcium overload conditions. The leading compound C-2h has been shown the most promising effects on all analyzed parameters. Thus, these compounds can be regarded as promising candidates for the design of multi-target disease-modifying drugs for treatment of AD and/or similar neuropathologies.

Neurodegenerative diseases, in particular, Alzheimer’s disease (AD), represent a prominent social and medical problem, because of the progressively increasing number of patients, considerable economic losses associated with medical attendance, and the lack of effective therapy^1^,^2^,^3^. The drugs used currently are mainly symptomatic; they partly restore the lost cognitive functions by activating some neurotransmitter systems. Despite the enormous effort and financial expenditures for the search for novel effective agents for treating Alzheimer disease, not a single drug against this disease has entered the market in the last 11 years^4^.

The key challenge is in the fact that the large part of developed drugs are directed to one target, which is chosen in accordance with the existing theories of AD pathogenesis. First of all, this is so-called amyloid hypothesis, which relates progression of the disease to the increased production of the β-amyloid (Aβ) peptide, and the tau-hypothesis, which implies the key role of aggregation of hyperphosphorylated tau-protein with accompanied destabilization of microtubules^5^.

The multifactor nature of AD is commonly recognized, implying the involvement a number of neurobiological targets in the initiation and development of this neurodegenerative disease. It includes different enzymes of neurotransmitter metabolism, CNS receptors and ionic channels involved in signal transduction, mitochondrial systems, inflammatory process etc. In this context, the concept of multitarget drugs having an integrated action on a number of biological targets involved in pathogenesis of the disease currently appears to be highly promising in the design of drugs for treating AD^6^,^7^,^8^. It can be expected that these drugs would be able not only to compensate or restore the lost cognitive functions, but also to suppress further development of the neurodegenerative process^9^,^10^,^11^,^12^.

It is known that the key neurobiological aspects affected by the AD development are the cholinergic and glutamatergic neurotransmitter systems^13^,^14^,^15^,^16^, microtubules system of intracellular transport, and brain mitochondria^17^. The search for the drugs that can compensate or restore the lost functions of these neurotransmitter systems was initially regarded as the most obvious drug design strategy for treating AD. The cholinesterase inhibitors, donepezil, rivastigmine, galantamine, and memantine (a low-affinity noncompetitive NMDA receptor antagonist) are so far the main therapeutic agents for treating this disease^18^,^19^. Combined application of these agents in the therapy of AD is actively employed^20^,^21^,^22^.

An important trend in the rational design of drugs acting on the pathogenesis of the disease comprises efforts to stabilize the intracellular transport provided by the system of microtubules. It was found that AD is associated with pathological hyperphosphorylation of the τ-protein; the aggregated forms of the protein form neurofibrillary tangles, a typical pathomorphological marker of AD, which gives rise to multiple neuronal disorders^23^. Recently, it was found that particularly the total content of the tau-protein and the content of phospho-tau are the most reliable markers of the development of AD and mild cognitive impairments associated with AD. Among all plasma biomarkers analyzed, only the total tau was significantly associated with AD. CSF Aβ42, total tau, and phospho-tau also distinguished the mild cognitive impairment due to AD from stable MCI^24^. Our previous data showed that polymerization of tubulin (Tb) and microtubule-associated proteins (MAP) isolated from postmortem brain samples of AD patients led to a decrease in polymerization and generation of abnormal tangled and bundled microtubules (MT)^25^. On the other hand, we have shown that some acetylcholinesterase inhibitors, in particular amiridine, can restore the impaired structure of Tb-MAPs MT from an AD-affected brain^26^. In general, it appears that agents which stimulate polymerization of tubulin to microtubules with normal structure can be considered as a promising drug-like candidates for AD treatment^27^,^28^,^29^.

The key strategy of our study was to develop multiligand agents that could exert synergistic action on several various pathogenetic targets, resulting in considerable enhancement of the overall pharmacological effect, and to provide these drugs with both cognitive-stimulating and disease-modifying action. It is believed that the development of a single chemical molecule able to act simultaneously on multiple pathogenetic units of the disease can offer additional advantages over combinations of several drugs as regards both the optimal ADMET profile and reduction of the risk of adverse events caused by interaction of particular drug components^30^,^31^.

It should be noted that the concept of multitarget agents supposes the focused design of structures that simultaneously interact with number of principal targets involved in disease development but does not mean that the interaction with each target would be characterized by the highest efficacy. For instance, the inhibitors of AChE with strongest binding parameters are not drugs but toxic agents; the high-affinity ligands of NMDA receptors, such as MK-801 possess neurotoxic but not neuroprotective activity, and so on. So it is expected that the most promising multitarget agents should have the optimal combination of activities towards number of key targets with moderate binding (or inhibition) properties for each target, yet the optimal balance of inhibitory ability against each target in AD remains unknown. From this standpoint in the present work we tried to design the compounds that possess simultaneously the properties of blockers of NMDA-receptors, inhibitors of enzymes of cholinesterase family, modulators of mitochondrial functions and tubulin polymerization system stabilizers.

For this purpose, we synthesized several groups of novel compounds combining in one molecule several pharmacophore moieties of specific ligands whose biological targets are known to be involved in AD pathogenesis^32^,^33^. In the assembly of hybrid polyfunctional molecules, we used memantine (1-amino-3,5-dimethyladamantane hydrochloride) and its aminoadamantane derivative amantadine as the first pharmacophore and carbazole and tetrahydrocarbazole derivatives as the second pharmacophore (Fig. 1).

Carbazoles and tetrahydrocarbazoles are known to possess a broad range of biological activity. The carbazole skeleton is the key structural motif of many synthetic and naturally occurring biologically active compounds^34^. In recent years, promising compounds for the design of disease-modifying drugs for the therapy of AD have been found among carbazole derivatives. In particular, aminotetrahydrocarbazoles are able to stimulate neurogenesis and to stabilize the endoplasmic reticulum (ER) calcium homeostasis by attenuating the FAD-PS1 mediated exaggerated ER calcium release. These compounds can also improve the mitochondrial function measured by increased mitochondrial membrane potential and lower the Aβ peptide production by decreasing the cleavage of amyloid precursor protein (APP) by β-secretase, without notably affecting α- and γ-secretase cleavage activities^35^,^36^,^37^. The synthesis of compounds combining two pharmacophores, aminoadamantane and carbazole ones, linked by a 2-hydroxypropylene spacer (Fig. 1) was reported in our previous papers^32^,^33^,^38^.

Here we studied the action of conjugates С-1 and С-2 on the key targets of the cholinergic and glutamatergic systems, in particular, acetylcholinesterase (EC 3.1.1.7, AChE), butyrylcholinesterase (EC 3.1.1.8, BChE), and carboxylesterase, which is an enzyme structurally related to cholinesterases (EC 3.1.1.1, CaE), as well as on binding to the intrachannel and allosteric (ifenprodil-binding) sites of NMDA receptors and on the microtubule assembly from tubulin in the *in vitro* system. For the most active compounds with the ability to influence some mitochondrial parameters^38^, their effects on cellular neurotoxicity were evaluated.

## Results

### The inhibitory activity of the conjugates C-1 and C-2 towards human erythrocyte AChE, equine serum BChE, and porcine liver CaE

All conjugates of aminoadamantanes with carbazole derivatives were tested as AChE, BChE, and CaE inhibitors. It is known that inhibition of CaE by anticholinesterase compounds can lead to undesirable drug-drug interactions^39^. We used human erythrocyte AChE, equine serum BChE, and porcine liver CaE. It was shown previously that the two last-mentioned enzymes have a high identity to the human enzymes^40^,^41^. The inhibitory activity was characterized as the percentage of inhibition at 20 μM or as the IC_50_ value, i.e., the inhibitor concentration required to reduce the enzyme activity by 50%. As the reference compounds, we used the basic pharmacophores: memantine, amantadine, and carbazole. Bis-4-nitrophenyl phosphate (BNPP), which is a selective CaE inhibitor, and tacrine, which is an effective AChE and BChE inhibitor, were used as positive controls in the study of enzyme inhibition.

**Figure 1:**
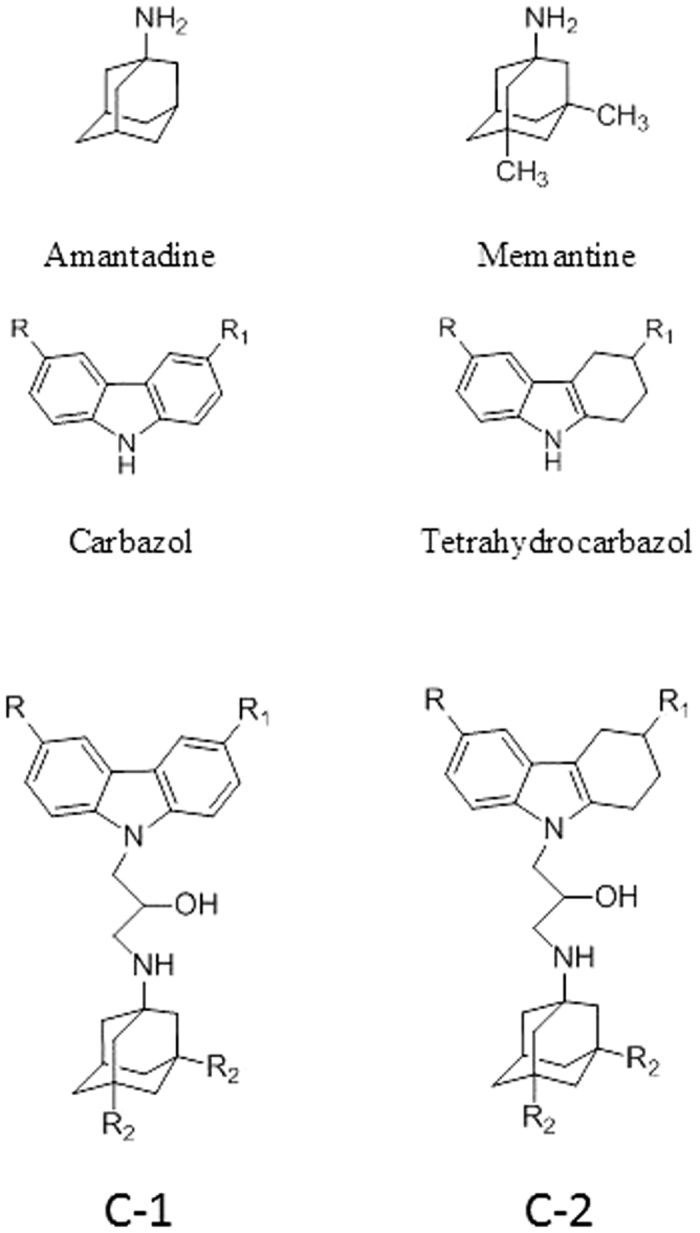
Structures of Amantadine, Memantine, Carbazole and Tetrahydrocarbazole and the studied conjugates of carbazoles (C-1) and tetrahydrocarbazoles (C-2); R_2_ = CH_3_ – memantine derivatives, R_2_ = H – amantadine derivatives.

The results, which are summarized in Table 1, indicate that all conjugates of aminoadamantanes with carbazole derivatives (Fig. 1) inhibit AChE and CaE very weakly but have fairly high inhibitory activity towards BChE. All compounds demonstrate the micromolar activity and very high selectivity to BChE. They all are much more potent inhibitors that the reference compounds, memantine, amantadine, and carbazole (Table 1).

Among all of the studied conjugates with the carbazole moiety, both containing amantadine and memantine as the second pharmacophore (C-1a–C-1f), the highest inhibitory activity is inherent in compounds C-1a and C-1b with R = R_1_ = H, the memantine derivative C-1b being twice as active (IC_50_ = 7.6 ± 0.3 μM) as the amantadine derivative (IC_50_ = 15.9 ± 1.0 μM). The other carbazole-containing conjugates also selectively inhibited BChE, but they were less active, with IC_50_ being in the 20–55 μM range.

The replacement of the carbazole pharmacophore (compounds C-1a–C-1f) by a tetrahydrocarbazole one (C-2a–C-2h) generally results in higher anti-BChE activity of the conjugate, except for compounds (С-2c) and (C-2d) with R = CH_3_, R_1_ = H, which retain lower inhibitory activities (Table 1). The other representatives of conjugates С-2 efficiently inhibit BChE, with IC_50_ values varying over a relatively narrow range, from 5.43 (C-2h) to 9.17 μM (C-2е). No significant difference was observed between the amantadine and memantine derivatives. The unsubstituted derivative C-2b (R = R_1_ = H) and fluorinated derivative C-2h (R = F, R_1_ = H) were most active among the tetrahydrocarbazoles and had IC_50_ = 6.13 ± 0.40 and 5.43 ± 0.39 μM, respectively.

**Figure 2:**
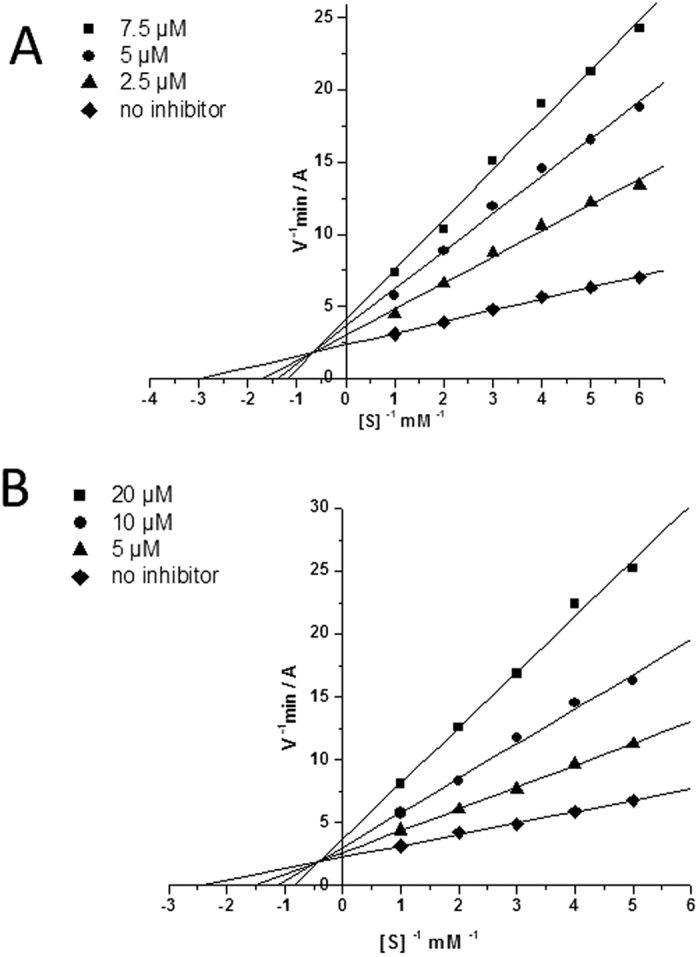
Steady state inhibition of BChE by compounds C-1b (**A**) and C-2g (**B**). Lineweaver-Burk reciprocal plots of initial velocity and substrate concentrations in the presence of inhibitors C-1b and C-2g, (three concentrations) and their absence are presented. The plots A and B show mixed-type inhibition.

The mechanism of inhibitory action of aminoadamantane conjugates with carbazole derivatives was illustrated in relation to two active compounds, C-1b and C-2g. The graphical analysis of the kinetic data on BChE inhibition by C-1b and C-2g in the Lineweaver–Burk double-reciprocal plot (Fig. 2) attests to a mixed type of inhibition, the inhibition constants being *K*_*i*_ = 2.11 ± 0.17 μM (competitive component) and *αK*_*i*_ = 8.48 ± 0.38 μM (noncompetitive component) for C-1b and *K*_*i*_ = 4.62 ± 0.23 μM (competitive component) and *αK*_*i*_ = 23.1 ± 2.7 μM (noncompetitive component) for C-2g.

### Molecular modeling

The nature of high inhibitory activity and selectivity of aminoadamantane–carbazole conjugates towards BChE was assessed by molecular docking of the compounds to BChE active site. Autodock 4.2 and Vina results were in very good agreement.

**Figure 3:**
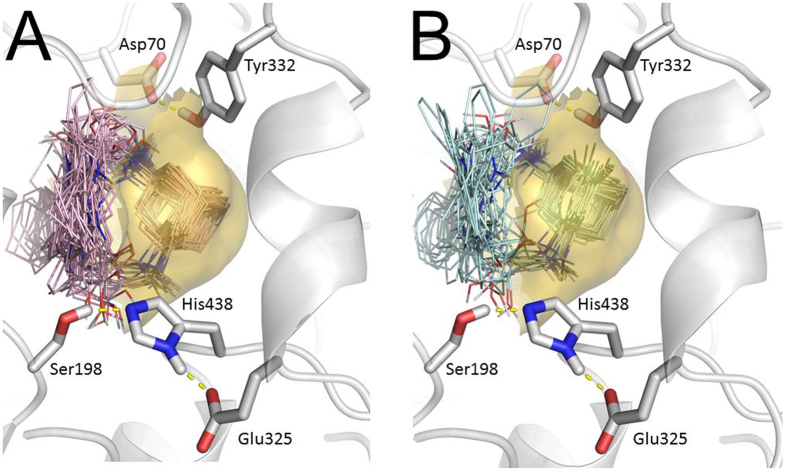
Binding positions obtained in 256 LGA runs, with respect of clusterization for compounds with amantadine (**A**) and memantine (**B**) fragments. Transparent surface shows a part of the BChE gorge engulfing these fragments. Catalytic triad of BChE Ser198•His438•Glu325 is shown in foreground; the residues of the PAS Asp70 and Tyr332 are also shown at the top.

Figure 3 shows the docked poses obtained during 256 runs of Lamarckian Genetic Algorithm (LGA) with respect of clusterization for compounds with amantadine (A) and memantine (B) moieties. Both amantadine and memantine moieties occupy the cavity in the BChE active site gorge below the peripheral anionic site (PAS, delimited by Asp 70 and Tyr332) and above the catalytic triad. Considering all binding poses obtained during molecular docking procedure, it can be noticed that the positions of amantadine and memantine moieties are very stable, while the positions of the linker and the carbazole part are highly variable.

**Figure 4:**
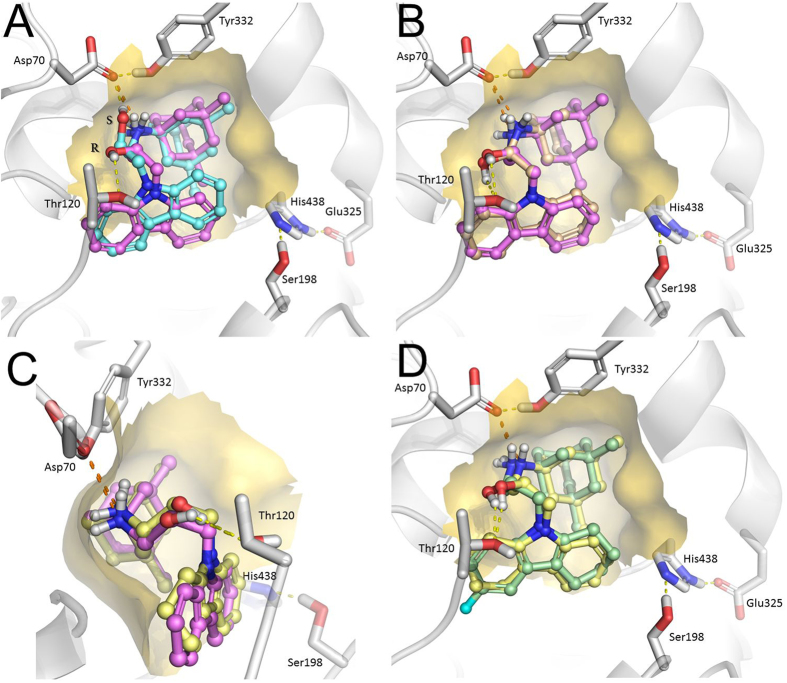
Molecular docking of conjugates C-1 and C-2 to the BChE active site. Dashed lines are showing hydrogen bonds (yellow) and salt bridges (orange). (**A**) Binding positions of R- (magenta) and S- (cyan) enantiomers of compound C-1b. (**B**) Overlay of compounds C-1a (peach) and C-1b (magenta) differing by amantadine and memantine fragments. (**C**) Overlay of compounds C-1b (magenta) and C-2b (yellow) differing by carbazole and tetrahydrocarbazole fragments. A side view comparing to panels A and B, bending of the ligands in the BChE gorge could be seen. As opposite to the Fig. 3, the view is reverse and the catalytic triad is on the background. (**D**) Overlay of compounds C-2b (yellow) and C-2h (green) differing by presence in the latter fluorine atom in the tetrahydrocarbazole ring, which causes flip of the ring.

For the subsequent analysis of the molecular docking results, only positions with the best binding affinities were used. The docked positions of conjugates of aminoadamantanes with carbazole derivatives С-1 and С-2 are presented in Fig. 4А–D.

Figure 4 shows that the strongest binding is secured by the salt bridge between the positively charged amino group and Asp70 side chain of the BChE PAS. Another specific interaction of the ligands with BChE gorge residues is the hydrogen bond formed by the hydroxyl group of 2-hydroxypropylene spacer. Compounds have a chiral center at the point of attachment; however, the results of molecular docking (Fig. 4A) suggest that the difference between the binding modes of the two enantiomers is insignificant, as it does not affect the overall position of the ligand in the gorge. The hydroxyl groups of the two enantiomers are hydrogen-bonded to different amino acid residues (Fig. 4A): Thr120 for the *R*-isomer and Asp70 for the *S*-isomer. No other differences were found. Owing to this similarity between the docked positions of the enantiomers, the docking results for only *R*-enantiomers of the inhibitors are described below.

According to Fig. 3, the positions of the amantadine and memantine groups are generally very similar; thus, these substituents do not cause significant changes in the ligand position (Fig. 4B for compounds C-1a and C-1b). Similarly, the position of the ligand does not change as the carbazole group is replaced by the tetrahydrocarbazole one (Fig. 4C); however, introduction of a halogen substituent, even as small as a fluorine atom, induces a flip of the carbazole group (Fig. 4D) to relieve the steric strain.

### Radioligand study of the interaction of compounds with NMDA-receptor binding sites

The radioligand assay was used to study binding of conjugates С-1 and C-2 to various modulating sites of NMDA receptors. The competition of substances with [^3^H]MK-801 (dizocilpine) was used to determine the ability of compounds to interact with the whole pool of NMDA receptors, while the competition with [^3^H]ifenprodil attested to the ability of compounds to interact with the type of NMDA receptors containing NR2B subunits. The latter feature is of particular interest, because antagonists of NR2B-containing NMDA-receptors were recently found to possess a substantial therapeutic potential^42^,^43^.

Ifenprodil is known also to interact with several other receptors, such as α1 adrenergic^44^, serotonin^45^ and σ receptors^46^. However, since we used a membrane preparation of rat cerebral hippocampus having the greatest density of NR2B-containing NMDA receptors^47^ and a low concentration of [^3^H] ifeprodil (5 nM), according with its high affinity for NR2B^48^,^49^,^50^, it is possible to neglect the contribution of ifenprodil binding to somewhat less sensitive serotonin and α1 adrenergic receptors^44^,^51^. As for the σ receptors which also bind ifenprodil in nanomolar concentrations, in the used hippocampus membrane fraction, σ receptor density is significantly less than that of NMDA-receptors^52^. All this gives reason to believe that our primary screening results reflect the predominant binding of the test compounds to NR2B-containing NMDA receptors.

The ability to block the receptor sites was estimated by the IC_50_ value, which corresponds to the compound concentration sufficient for decreasing the level of the bound labeled specific ligand by 50%. The results summarized in Table 2 indicate that all aminoadamantane–carbazole conjugates (C-1) have a moderate affinity to the intrachannel (MK-801-binding) site of the NMDA receptor, except for compound С-1f, which contains two chlorine atoms in the carbazole moiety (IC_50_ = 22.4 μM). Conjugates C-1 are active to different extents towards the ifenprodil-binding site, this activity being more dependent on substituents in the carbazole moiety than on the structure of the aminoadamantane moiety. The highest ifenprodil-binding site blocking activity was found for combinations of unsubstituted carbazole and amantadine moieties (С-1a) (IC_50_ = 8.2 μM) and dichlorocarbazole and memantine moieties (C-1f) (IC_50_ = 14.6 μM). Furthermore, compound С-1а blocked only the ifenprodil-binding site, while С-1f blocked also the MK-801-binding site, although less efficiently than it blocked the ifenprodil site (IC_50_ = 22.4 μM),

Unlike carbazole conjugates, some tetrahydrocarbazole conjugates with aminoadamantanes (С-2) were found to exhibit high affinity to the NMDA receptor intrachannel (MK-801-binding) site (compound С-2b), ifenprodil-binding site (C-2e), and both sites (С-2f, С-2h, Table 2). Comparison of analogous conjugates formed by carbazoles and tetrahydrocarbazoles shows that whereas in the case of carbazoles, compound С-1а devoid of substituents in the carbazole moiety has the highest activity towards the ifenprodil-binding site, analogous tetrahydrocarbazole derivative С-2а (R = R_1_ = H) is inactive, while compound C-2e containing two methyl groups in the tetrahydrocarbazole moiety is active, the activities of С-1a and C-2e being virtually equal.

Among tetrahydrocarbazole derivatives, C-2f (IC_50_ = 19.5 and 10.3 μM, respectively) and C-2h (IC_50_ = 27.4 and 10.4 μM, respectively) are most active towards both binding sites; compound C-2b is selective towards MK-801-binding site (IC_50_ = 14.2 μM); and compound С-2е is selective towards the ifenprodil-binding site (IC_50_ = 8.1 μM).

It is noteworthy that among all of the conjugates studied, the activity towards the MK-801-binding site was found only for conjugates containing memantine rather than amantadine. The activity towards the ifenprodil-binding site is a characteristic distinctive feature of the new conjugates, as the initial pharmacophores, carbazoles and aminoadamantanes, do not possess this activity.

### Influence of the compounds on the microtubule assembly and structure

The primary screening for the effect on tubulin polymerization *in vitro* was performed using a commercial tubulin in the presence of 0.1 mM of the carbazole–aminoadamantane conjugates. In blank probes, tubulin (40 μM) polymerization in the presence of 10% DMSO (as in the case of test probes) leads to absorbance increase after a rather long (80–120 minutes) lag period. The primary screening allowed us to reveal the compounds that substantially reduced the lag period and increased the polymerization rate and the amount of aggregates formed. The results are summarized in Table 3 as the maximum rate of absorbance increase for a tubulin- microtubules suspension normalized to the blank value.

For both carbazole and tetrahydrocarbazole conjugates, no regular patterns and significant dependence on the second pharmacophore, either amantadine or memantine, was found. The introduction of chloro- or bromo- substituent’s into the carbazole pharmacophore results in almost complete loss of the ability to stimulate tubulin polymerization (С-1с–C-1f). Meanwhile, the introduction of fluorine into the tetrahydrocarbazole pharmacophore is accompanied by a considerable enhancement of the tubulin polymerization-stimulating effect (С-2g and C-2h) (Table 3).

**Figure 5:**
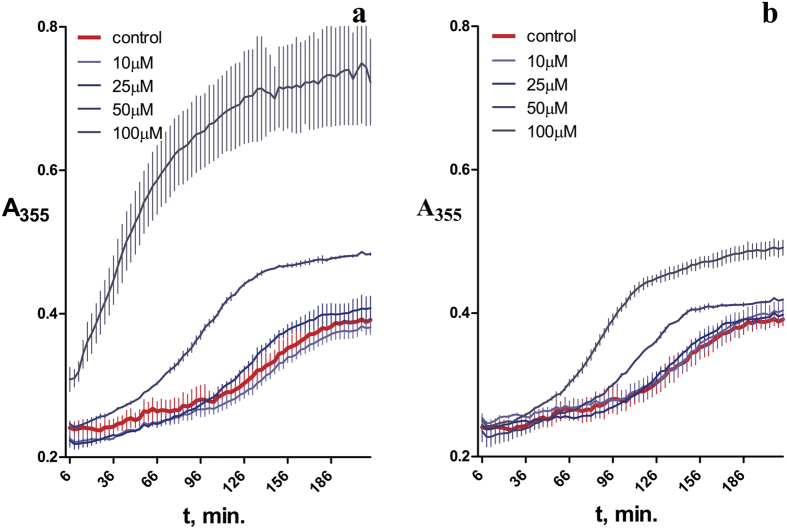
Influence of compounds C-2h (**a**) and C-1a (**b**) on polymerization of tubulin to microtubules: kinetics of absorbance increase at λ 355 nm with tubulin polymerization to microtubules.

The most pronounced ability to promote the assembly of microtubules was found for carbazole derivatives C-1a and C-1b (R = R_1_ = H), tetrahydrocarbazole derivatives C-2d (R = CH_3_, R_1_ = H), and fluorine-containing compound C-2h (R = F, R_1_ = H), which, being present in 0.1 mM concentration, increased the tubulin polymerization rate to 192 ± 14, 249 ± 16, 182 ± 16, and 330 ± 40%, respectively. In the case of the most active fluorinated derivative (C-2h), a concentration dependence of this effect was shown, with simultaneous shortening of the lag period and increase in the maximum absorbance of tubulin suspensions with respect to the blank (Fig. 5), which implies a higher content of polymerized tubulin.

**Figure 6:**
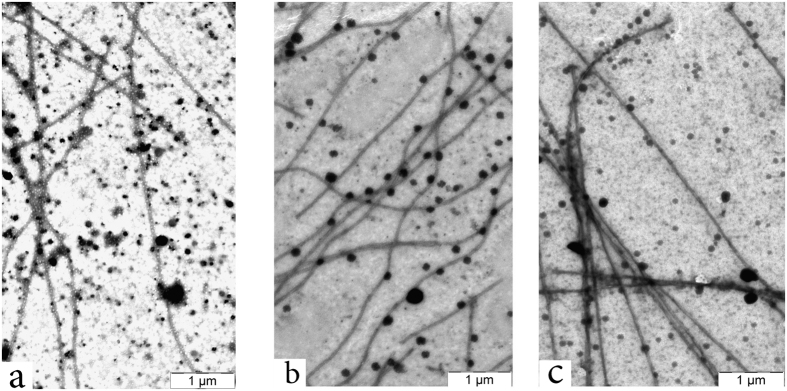
Microtubules formed in the presence of (**a**) only vehicle (control), (**b**) 100 μM of compound С-2h, (**c**) 100 μM of compound С-1a. Electron microscopy (x10, 000) was performed after *in vitro* polymerization of tubulin.

An electron microscopy examination of the resulting tubulin polymers using the negative staining technique demonstrated that in the presence of all active compounds, in particularly of C-2h and C-1a, microtubules of the normal structure are formed (Fig. 6A), i.e., long and straight microtubules of a regular shape, arranged in pairs, similar to the microtubules of the blank experiment (Fig. 6B).

### Evaluation of the cytoprotective properties of the compounds

**Figure 7:**
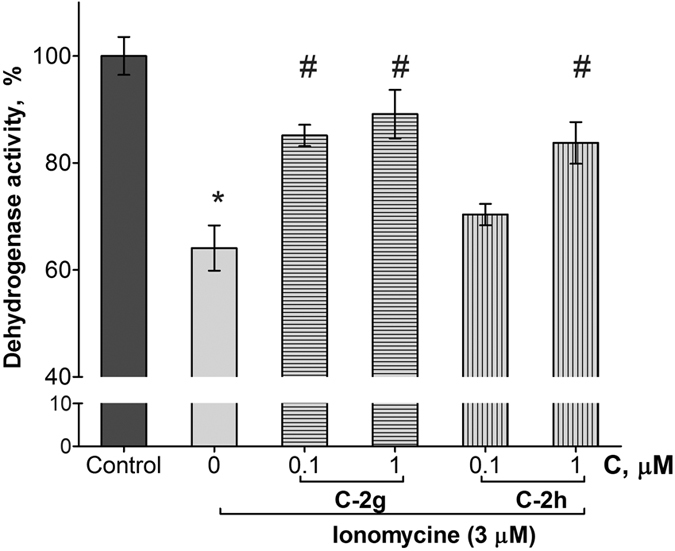
Influence of compounds C-2g and C-2h on ionomycine-induced toxicity of the human neuroblastoma cell line, SH-SY5Y. Incubation of cells with the test compounds and ionomycine was during 24 hours. Then dehydrogenase activity was measured by MTT test. Control samples contained DMSO (≤1%) instead of the test compounds. Ionomycine concentration was 3 μM. *Indicates *p* < 0.05 compared to the control cultures (T-test), ^#^*p* < 0.05 compared to the ionomycine (ANOVA-test).

In order to evaluate the potential neuroprotective action of new compounds, we studied the effect of the most promising fluorinated compounds C-2g and C-2h on the ionomycin-induced cytotoxicity in relation to the SH-SY5Y neuroblastoma cell culture. As can be seen in Fig. 7, the incubation for 24 hours with 3 μМ ionomycin reduces the neuroblastoma cell survival by 36 ± 4%. The presence of 1 μМ of compounds C-2h and C-2g efficiently suppresses the development of ionomycin-induced cytotoxicity and cell survival increases to 84 ± 4% and 89 ± 4%, respectively. It should be noted that in the absence of ionomycin compounds C-2g and C-2h in the studied concentrations did not affect the MTT test parameters (i.e., cell survival did not differ significantly from 100%).

## Discussion

The key goal of this work was a focused design of original multitarget compounds as drug candidates for the therapy of neurodegenerative diseases; first of all, AD, the pathogenesis of which is associated in particularly with the impairment of cholinergic and glutamatergic neurotransmitter systems and with the disorder of microtubule system. We made use of the idea of development of hybrid templates comprising pharmacophores of specific ligands of the key targets involved in the pathogenesis of such diseases, namely, aminoadamantane and carbazole derivatives.

The primary screening of the newly synthesized series of compounds was carried out using the integrated screening system comprising measurement of the inhibitory activity towards AChE, BChE, and CaE, determination of binding to two sites of the NMDA-subtype of glutamate receptors by radioligand assay, and elucidation of the influence of new compounds on the *in vitro* microtubule assembly from tubulin. Memantine, amantadine, and carbazole were used as the reference compounds. In order to identify the hit compounds in each screening block, a more detailed investigation on the comparative analysis of interaction with various types of cholinesterases was performed by enzyme kinetics and molecular docking methods. The IC_50_ values for the most effective glutamate receptor modulators were determined, and the structure of the tubulin polymers formed in the presence of compounds that exhibited the ability to promote this process was examined by electron microscopy, and the neuroprotective activity of two promising compounds was tested on the calcium overload cellular model.

The results presented in Table 1 indicate that, irrespective of the combination of pharmacophores (aminoadamantane and carbazole ones), all of the conjugates we studied inhibited very weakly AChE and CaE, but showed fairly high inhibitory activity towards BChE. That is, unlike reference compounds, memantine, amantadine, and carbazole, which almost do not inhibit any of these esterases, the novel conjugates are selective BChE inhibitors, with an inhibitory action being manifested in the micromolar range. The inhibition is reversible and occurs by a mixed-type mechanism (Fig. 2). Very low activity of conjugates C-1 and C-2 against AChE indicates that these compounds will not cause unwanted side effects as inherent AChE inhibitors, and lacking inhibitor activity against CaE suggests they will not cause adverse drug-drug interactions.

The results of kinetic measurements are confirmed by the data of molecular docking, which demonstrated that the amantadine and memantine moieties of the conjugates are located in the cavity of the BChE active site gorge below the peripheral anionic site and above the catalytic triad, and the conjugate binding is mainly due to the salt bridge between the positively charged secondary amino group of the ligand and the Asp70 side chain. One more specific interaction of the ligand with BChE is hydrogen bonding of the hydroxyl group of 2-hydroxypropylene spacer. Furthermore, the molecular docking results are in good agreement with the measured anti-BChE activity of the conjugates, which is within one order of magnitude for all of the compounds. Despite the differences between the structures of substituents, the ligand positions are very close to one another (almost identical) in all cases and fit well into the BChE gorge.

As regards the very weak inhibition of AChE by these conjugates, it is known that the AChE active site gorge is much narrower than that of BChE. Indeed, the cavity in the BChE active site gorge capable of accommodating an amantadine/memantine moiety is formed, in particular, by a small side group of the Ala328 residue, which corresponds to markedly larger Tyr337 residue in AChE; this precludes accommodation of the bulky and rigid amantadine/memantine moiety in this part of the AChE gorge. Also, a typical feature of the AChE active site gorge is the presence of so-called bottleneck formed by Tyr341 and Tyr124 residues, which control the downward movement of ligands to the active site and which can be impassable for the most bulky and rigid groups^53^. As in many other cases, these differences between the active site gorge structures in AChE and BChE dictate the selective inhibition of these related enzymes^53^,^54^. It is noteworthy that selective inhibition of BChE compared with AChE was also found for adamantyl derivatives known from the literature^55^,^56^.

In a healthy brain, acetylcholine is mainly hydrolyzed by AChE, whereas BChE plays an auxiliary role. However, in AD patients, the AChE activity decreases, whereas the activity of BChE gradually increases. This leads to a higher role of BChE as a therapeutic target for reducing the cholinergic deficiency present in AD patients^57^,^58^,^59^. As shown in recent studies, selective BChE inhibitors increase the acetylcholine level in brain and improve the cognitive functions in aged rodents, without inducing the classical side effects of AChE inhibitors^60^. Selective BChE inhibitors were shown to ameliorate the cognitive dysfunction induced by Aβ peptide in mice. BChE inhibition, as well as AChE inhibition, is now considered as a viable therapeutic strategy for cognitive dysfunction in AD^57^. Moreover, in AD case, numerous Aβ plaques are associated with BChE activity, which was also shown in a 5xFAD mouse model of AD. Knock-out of BChE (5xFAD/BChE-KO) led to about 70% diminished fibrillar Aβ plaque deposition. This suggests that the lack of BChE reduces the deposition of fibrillar Aβ in AD^61^,^62^,^63^,^64^, thus demonstrating that diminished BChE activity could prove beneficial as a curative approach to AD. These new data obtained on animal models along with analysis of clinical investigations^65^ indicate that BChE is a promising target for AD treatment and that inhibiting this enzyme would be of clinical value. High activity and BChE selectivity of conjugates C-1a, C-1b, C-2a, С-2b, С-2e, С-2f, С-2g, and C-2h are of interest for the design of drugs for safer therapy of AD.

The use of NMDA receptor antagonists for treating AD and other neurodegenerative diseases is currently restricted to memantine, a low-affinity intrachannel MK-801-binding site blocker^66^, which shows a good potential dependence of the ligand binding. No other blockers, apart from nitromemantine, functioning similarly to memantine and having therapeutic prospects have currently been registered^67^. It is known that NMDA receptors play an important role in the mammalian brain, being involved not only in the brain development and cognitive processes, but also in the development of some acute and chronic neurodegenerative diseases, including stroke, AD, and depression. An important role in implementation of the neurophysiological functions of NMDA receptors belongs to so-called NR2В subunits. Blockage of the NR2B subunits was shown to be responsible for the cognitive-stimulating and neuroprotective action of drugs, and also to mitigate the neuropathic pain, and to prevent convulsions^43^,^68^,^69^. The search for effective antagonists of NMDA receptors containing NR2B subunits is an important trend in the search for new neuroprotective agents^43^,^70^,^71^,^72^. The active compounds found in this study (C-1a and C-2e) are of considerable interest as a new original chemotype of ifenprodil-binding site blockers for NMDA-receptors.

Currently, there are very few known NMDA receptor blockers that are capable of blocking simultaneously the MK-801-binding site and ifenprodil-binding site^50^. Therefore, the ability to block both the allosteric (ifenprodil-binding) site of NMDA receptors and the intrachannel (MK-801-specific) binding site found for a number of synthesized compounds (C-1f, C-2f, and C-2h) appears to be a valuable result boosting the expected therapeutic potential of these compounds for treating AD, stroke, and/or depressions.

The simultaneous ability to selectively inhibit BChE (without noticeable suppression of the AChE activity) and block the ifenprodil-binding site of NMDA receptors found for some new compounds (С-1a, C-2e, C-2f, and C-2h) may substantially enhance the general therapeutic effect in the treatment of AD and as well as related diseases.

Among the new conjugates, aminoadamantane and carbazole derivatives described in this study, some compounds were found to accelerate tubulin polymerization, increase the amount of polymerized tubulin to microtubules of a normal structure. First of all, these are compounds С-1a, С-1b, and С-2d and the most active microtubule stabilizer, С-2h. The ability to potentiate the assembly of microtubules of a normal structure is of particular interest for the development of the new, disease-modifying type of drugs. Currently, several agents with this type of pharmacological activity are under clinical trials, in particular, these are the taxoid TPI-287, Epothilone B (phase I clinical trials), and NAP peptide or davunetide (phase II/III clinical trials)^73^. As abovementioned, microtubule structure disturbance, suppression of tubulin polymerization, and destabilization of microtubules are distinctive features of AD and some other neurodegenerative diseases. The destabilization of axonal microtubules is considered to account for one of the determining and early signs of neurodegeneration, viz., the degradation of axons. The neuron axonal microtubules are most stable as compared with the microtubules of other cells; however, they still occur in dynamic equilibrium with tubulin dimers, and excessive stabilization may result in neuropathy, as was shown for paclitaxel^74^. Therefore, it appears pertinent to look for moderate stabilizers of microtubules, and compounds potentiating the assembly of microtubules identified in the present study can be assigned to this particular class.

An important contribution to the neuroprotective potential can also be made by the ability of the compounds (C-2g) to diminish, in the dose-dependent manner, the calcium-induced swelling of isolated mitochondria, which may indicate the properties of this compound and its derivatives as inhibitors of mitochondrial permeability transition^38^. This specific process plays a key role in the cell death cascade in both the physiological cell apoptosis and various neurotoxic scenarios (glutamate excitotoxicity, amyloid toxicity, *etc*.). A condition absolutely necessary for the induction of this process is a considerable increase in the calcium concentration. Therefore, we studied the possible neuroprotective potential of compound C-2g and its memantine-containing analogue C-2h, the lead compound which exhibited multitarget activity in this study, on the ionomycin-induced cytotoxicity for the SH-SY5Y neuroblastoma cell culture.

In the study of the neuroprotective effect on the cellular model of neurodegeneration both compounds efficiently protected cells from death at ionomycin-induced calcium overload. This model reflects, at some extent, the major cause of cell death in hypoxic-ischemic injury, upon the β-amyloid action, or under excitotoxicity conditions. The results suggest neuroprotective action of compounds С-2g and С-2h under various conditions of neurotoxicity associated with the neuronal calcium overload and, at least one possible mechanism of this effect is the mitochondrial permeability transition inhibition.

## Conclusions

The strategy of focused synthesis of multipharmacophore compounds was utilized to prepare and study a novel group of compounds based on aminoadamantane/carbazole conjugates. Compounds of this class were found to act on a group of biological targets that play an important role in the development of AD and other neurodegenerative diseases related to the disorder of cholinergic and glutamatergic neurotransmitters systems, impairment of the microtubule system and mitochondrial dysfunction. First of all, these compounds selectively inhibit BChE, block NMDA receptors containing NR2B subunits, while maintaining the functions of MK-801-binding site blockers. They also possess the properties of stabilizers of microtubules and the ability to protect nerve cells from death at the calcium overload. The leading compound C-2h has shown the most promising results in all analyzed parameters. This allows us to consider these compounds as promising candidates for the design of multitarget disease-modifying drugs for treating AD and related neuronal pathologies.

## Materials and Methods

### Chemistry

The studied conjugates of aminoadamantanes and carbazole derivatives (Fig. 1) have been synthesized as described previously^32^,^33^,^38^.

### Biological assay

#### *In vitro* AChE, BChE, and CaE inhibition

Acetylcholinesterase (AChE, EC 3.1.1.7, from human erythrocyte), butyrylcholinesterase (BChE, EC 3.1.1.8, from equine serum), carboxylesterase (CaE, EC 3.1.1.1, from porcine liver), acetylthiocholine iodide (ATCh), butylthiocholine iodide (BTCh), 5,5′-dithiobis-(2-nitrobenzoic acid) (DTNB), and 4-nitrophenol acetate (4-NPA), were purchased from Sigma-Aldrich (Germany).

The AChE and BChE activities were measured by the method of Ellman and co-workers as described earlier^75^. The assay solution consisted of 0.1 M K/Na phosphate buffer (pH 7.5, 25 °C) with the addition of 0.33 mM DTNB, 0.02 unit/mL of AChE or BChE, and 1 mM of substrate (ATCh or BTCh, respectively). The assays were carried out with a blank containing all components except ATCh and BTCh in order to take account of a non-enzymatic reaction.

The activity of CaE was determined spectrophotometrically by the release of 4-nitrophenol at 405 nm^76^. The assay solution consisted of 0.1 M K/Na phosphate buffer (pH 8.0, 25 °C) with the addition of 1 mM 4-nitrophenyl acetate and 0.02 unit/mL of CaE. The assays were carried out with a blank containing all components except CaE.

The test compounds were dissolved in DMSO; the incubation mixture contained 2% of the solvent. Eight different concentrations of the test compounds in the range of 10^−11^–10^−4^ M were selected in order to obtain inhibition of AChE and BChE activity between 20% and 80%. The test compounds were added to the assay solution and pre-incubated at 25 °C with the enzymes for 10 min followed by the addition of the substrate. A parallel control was made for the assay solution with no inhibitor. Measurements were performed in a FLUOStar OPTIMA multifunctional reader (BMG Labtech, Germany). Each experiment was performed in triplicate. The results were expressed as the mean ± SEM. The reaction rates in the presence and absence of inhibitor were compared, and the percentage of residual enzyme activity due to the presence of test compounds was calculated. The IC_50_ (the concentration of inhibitor required to decrease the enzyme activity by 50%) values were determined graphically from inhibition curves (log inhibitor concentration vs percentage of residual enzyme activity) using the Origin 6.1 software.

#### Kinetic analysis of BChE inhibition. Determination of steady-state inhibition constants

To elucidate the inhibition mechanisms for the active compounds, the BChE residual activity was determined in the presence of three increasing concentrations of the test compounds and six decreasing concentrations of the substrates. The test compounds were pre-incubated with the enzymes at 25 °C for 10 min, followed by the addition of the substrates. Parallel controls were made to find the rate of hydrolysis of the same concentrations of substrates in the solutions with no inhibitor. The kinetic parameters of substrate hydrolysis were determined. Measurements were performed in a FLUOStar OPTIMA multifunctional reader (BMG Labtech, Germany). Each experiment was performed in triplicate. The results were fitted into Lineweaver-Burk double-reciprocal kinetic plots of 1/V versus 1/[S] and the values of inhibition constants *K*_*i*_ (competitive component) and *αK*_*i*_ (noncompetitive component) were calculated using the Origin 6.1 software.

#### Radioligand study of interaction of the compounds with NMDA-receptor binding sites

The effect of test compounds on the radioligand binding to NMDA receptors was determined by using a modified method, as reported earlier by Zhou L-M and co- workers^77^. Two radioactive ligands were used: [^3^H] MK-801 (dizocilpine) with a specific activity of 210 Ci/mmol, binding to all isolated NMDA receptors, and [^3^H] ifenprodil with a specific activity of 79 Ci/mmol, binding only to NMDA receptors containing the NR2B subunit^47^,^78^.

A hippocampal membrane specimen for radioligand analysis was prepared by the techniques described previously^79^. The obtained membrane pellet was resuspended in a work buffer (5 mM HEPES/4.5 mM Tris buffer, pH 7.6) in a ratio of 1:5 and stored in liquid nitrogen. The reaction mixture (the final volume of 0.5 ml) contained 200 μl of the working buffer, 50 μl of 50 nM radioligand solution, and 250 μl of the membrane suspension. Nonspecific binding was determined in the presence of 50 μl of 1 mM of unlabeled ligand.

For binding study, the reaction mixture was incubated at room temperature for 2 hours. After incubation, the samples were filtered through GF/B glass-fiber filters (Whatman), washed with the work buffer, dried, and transferred to scintillation vials to which 5 ml of scintillation fluid was added containing 4 g of diphenyloxazole (PPO), 0.2 g of diphenyloxazoil benzene (POPOP), and 1 liter of toluene. The radioactivity was determined in a TriCarb2800 TR scintillation counter (Perkin Elmer, Packard, USA) with counting efficiency of about 65%.

The effect of the test compounds on the binding of [^3^H] MK-801 and [^3^H] ifenprodil to rat hippocampal membranes was studied by adding 50 μl of the test compounds in the concentration range of 10^−8^–10^−3^ M to the incubation medium. By the results of inhibition, IC_50_ values were calculated for the test compounds using GraphPadPrism 4 Demo. In the cases where inhibition by the test compound in the 100 μM concentration did not exceed 50%, the value of IC_50_ was not determined (n/d).

#### Molecular modeling

For molecular modeling, the secondary amino group was considered ionized, which is supported by Marvin 14.9.1.0 (ChemAxon, http://www.chemaxon.com) p*K*_a_ estimation around 10. The ligands under consideration have a chiral center at the point of the hydroxyl group attachment, and both enantiomers were used for molecular modeling. The ligand geometries were optimized quantum-mechanically using Gamess-US package^80^, the B3LYP DFT method in the 6–31 G* basis set.

For molecular docking of the ligands into BChE, the PDB ID 1P0I^81^ structure was used. The protein structure was prepared, saturated with water molecules, and optimized using QM/MM method as reported previously^82^,^83^. The molecular docking with a Lamarckian Genetic Algorithm (LGA)^84^ was performed with the Autodock 4.2.6^85^ and AutoDock Vina 1.1.2 software^86^. The grid box for docking included the whole BChE active site and the gorge with 15 Å × 20.25 Å × 18 Å dimensions with grid spacing of 0.375 Å. The main of selected LGA parameters were 256 runs, 25 × 10^6^ evaluations, 27 × 10^4^ generations and population size of 300. For AutoDock Vina the same box and exhaustiveness 40 were used. The structural images were prepared with PyMOL (Schrödinger, LLC).

#### Tubulin polymerization

The assembly of tubulin into microtubules was carried out using pure tubulin from HTS- Tubulin polymerization assay kit (Cytoskeleton, Inc., Denver, USA). Tubulin polymerization assay was based on an adaptation of the original method of Shelanski *et al*. and Lee *et al*.^87^,^88^, who demonstrated that light is scattered by microtubules to an extent that is proportional to the concentration of microtubule polymer. The standard polymerization reaction contained 100 μl of a 4 mg/ml tubulin solution in 80 mM PIPES (pH 6.9), 0.5 mM EGTA, 2 mM MgCl_2_, and 1 mM GTP. Polymerization was monitored by observing the change in the absorbance on a Plate reader Victor (Perkin Elmer, Finland) at λ = 355 nm. The parameter V_max_ = (dA_355_/dt)^max^ normalized to control probes (%) was used to compare the action of the compounds. Electron microscopic monitoring was carried out on Carl Zeiss Libra 120 Electron Microscope (Carl Zeiss Meditec AG, Jena, Germany) at 120 kV using negative countstaining^89^.

#### Effect of compounds on Ionomycin-induced toxicity in SH-SY5Y neuroblastoma cells

##### 

The human neuroblastoma cell line SH-SY5Y was used for all experiments. The cells were cultured in a humidified, 5% (v/v) CO_2_-controlled environment at 37 °C, and grown in MEM/F12 culture medium supplemented with 15% fetal bovine serum (FBS), 100 U/mL penicillin, and 100 mg/mL streptomycin^90^.

The cell viability was evaluated as the dehydrogenase activity with the 3-(4,5-dimethylthiazol-2-yl)-2,5-diphenyltetrazolium bromide (MTT) assay^91^. Briefly, the SH-SY5Y cells were incubated with 0.1 and 1 μM of a test compound or an equal volume of the vehicle (<1% of the whole volume of the medium under the layer of cells) and 3 μM ionomycine for 24 h. The absorbance was measured at 570 nm using a Victor microplate reader (Perkin Elmer).

## Additional Information

**How to cite this article:** Bachurin, S. O. *et al*. Novel conjugates of aminoadamantanes with carbazole derivatives as potential multitarget agents for AD treatment. *Sci. Rep.* **7**, 45627; doi: 10.1038/srep45627 (2017).

**Publisher's note:** Springer Nature remains neutral with regard to jurisdictional claims in published maps and institutional affiliations.

## Figures and Tables

**Table 1:** Inhibitory activity (IC_50_, μM) of conjugates of aminoadamantanes with carbazole derivatives **C**-**1** and **C**-**2** (Fig. 1) towards AChE, BChE and CaE.

Compounds				IC_50_ (μM) ± SEM or (inhibition % at 20 μM)		
No	R	R_1_	R_2_	AChE	BChE	CaE
C-1a	H	H	H	>20 (2.8%)	15.9 ± 1.0	>20 (9.3%)
C-1b	H	H	CH_3_	n.a.	7.6 ± 0.3	>20 (10.5%)
C-1c	Br	Br	H	>20 (5.5%)	20.7 ± 0.2	>20 (25.3%)
C-1d	Br	Br	CH_3_	>20 (6.1%)	55.4 ± 4.9	n.a.
C-1e	CI	CI	H	>20 (13.1%)	23.7 ± 0.4	50.1 ± 4.8
C-1f	CI	CI	CH_3_	>20 (4.0%)	40.7 ± 3.2	n.a.
C-2a	H	H	H	>20 (4.6%)	7.29 ± 0.50	>20 (17.9%)
C-2b	H	H	CH_3_	>20 (5.1%)	6.13 ± 0.40	>20 (10.1%)
C-2c	CH_3_	H	H	>20 (3.9%)	20.02 ± 1.18	>20 (15.1%)
C-2d	CH_3_	H	CH_3_	>20 (2.2%)	33.0 ± 0.2	>20 (12.3%)
C-2e	CH_3_	CH_3_	H	>20 (7.1%)	9.17 ± 0.74	>20 (20.0%)
C-2f	CH_3_	CH_3_	CH_3_	>20 (7.3%)	8.24 ± 0.36	>20 (10.3%)
C-2g	F	H	H	>20 (4.9%)	8.66 ± 0.32	>20 (15.4%)
C-2h	F	H	CH_3_	>20 (8.8%)	5.43 ± 0.39	>20 (16.5%)
Memantine				>20 (1.3%)	>20 (13.7%)	>20 (3.0%)
Amantadine				>20 (3.4%)	>20 (6.9%)	>20 (3.2%)
Carbazole				>20 (1.8%)	>20 (16.1%)	>20 (9.4%)
Tacrine				0.60 ± 0.05	0.0290 ± 0.0002	n.a.
BNPP				n.a.	n.a.	1.80 ± 0.11

^*^n.a. = not active.

**Table 2:** The binding of conjugates of aminoadamantanes with carbazole derivatives **C**-**1** and **C**-**2** (Fig. 1) to МК-801 and ifenprodil binding sites of NMDA receptor.

Compounds				Binding characteristics of compounds			
No	R	R_1_	R_2_	% of [^3^H]МК-801 blockade at 100 μM of compound	[^3^H]МК-801, IC_50_, μM	% of [^3^H]ifenprodil blockade at 100 μM of compound	[^3^H]ifenprodil, IC_50_, μM
C-1a	H	H	H	83.5 ± 7.6	75.3 ± 6.9	74.2 ± 6.8	8.2 ± 0.8
C-1b	H	H	CH_3_	68.5 ± 5.4	59.1 ± 4.9	76.2 ± 6.6	21.3 ± 1.9
C-1c	Br	Br	H	55.1 ± 4.9	79.4 ± 7.5	85.3 ± 7.6	22.9 ± 1.8
C-1d	Br	Br	CH_3_	33.8 ± 3.5	>100	67.6 ± 5.8	31.5 ± 3.3
C-1e	CI	CI	H	28.3 ± 1.8	>100	70.6 ± 7.1	21.8 ± 1.8
C-1f	CI	CI	CH_3_	72.8 ± 6.8	22.4 ± 2.1	71.4 ± 5.7	14.6 ± 1.5
C-2a	H	H	H	32.3 ± 3.0	>100	71.6 ± 8.3	82.5 ± 9.3
C-2b	H	H	CH_3_	62.2 ± 6.3	14.2 ± 2.3	58.4 ± 5.6	65.5 ± 6.1
C-2c	CH_3_	H	H	0 ± 2.0	>100	51.6 ± 7.2	98.4 ± 6.9
C-2d	CH_3_	H	CH_3_	18.3 ± 2.0	>100	31.4 ± 3.1	>100
C-2e	CH_3_	CH_3_	H	29.3 ± 6.4	>100	67.2 ± 7.3	8.1 ± 1.7
C-2f	CH_3_	CH_3_	CH_3_	65.2 ± 5.3	19.5 ± 1.6	71.4 ± 6.5	10.3 ± 1.9
C-2g	F	H	H	0 ± 2.0	>100	48.4 ± 5.2	>100
C-2h	F	H	CH_3_	88.5 ± 9.0	27.4 ± 4.1	87.2 ± 7.3	10.4 ± 2.9
Memantine				100	1.36 ± 0.086	11.2 ± 1.1	>100
Amantadine				57.2 ± 5.5	35.5 ± 3.2	44.6 ± 4.6	>100

**Table 3:** Influence of conjugates of aminoadamantanes with carbazole derivatives C-1 and C-2 (Fig. 1) on tubulin polymerization.

Compounds				(dA_355_/dt)^max^, % (at 100 μM of compound)
No	R	R_1_	R_2_	100 ± 14
C-1a	H	H	H	192 ± 14
C-1b	H	H	CH_3_	249 ± 16
C-1c	Br	Br	H	124 ± 17
C-1d	Br	Br	CH_3_	104 ± 24
C-1e	CI	CI	H	100 ± 8
C-1f	CI	CI	CH_3_	101 ± 15
C-2a	H	H	H	155 ± 22
C-2b	H	H	CH_3_	127 ± 10
C-2c	CH_3_	H	H	152 ± 12
C-2d	CH_3_	H	CH_3_	182 ± 16
C-2e	CH_3_	CH_3_	H	158 ± 19
C-2f	CH_3_	CH_3_	CH_3_	133 ± 14
C-2g	F	H	H	164 ± 18
C-2h	F	H	CH_3_	330 ± 40
Memantine				89 ± 7
Amantadine				98 ± 16
Carbazole				123 ± 12
Paclitaxel (5 μM)				487 ± 32

Data presented as percent of maximum rate of 355 nm absorbance increase due to microtubules assembly. 100% - maximum rate of 355 nm absorbance in control probe with only vehicle (10% of DMSO).
